# Study on the Epidemiological Characteristics, Treatment Patterns, and Factors Influencing the Timeliness of Treatment in Head and Neck Squamous Cell Carcinoma (HNSCC) in Stages III and IV: Experience of a Mexican Hospital

**DOI:** 10.3390/jpm15050193

**Published:** 2025-05-09

**Authors:** Victor Manuel Oyervides Juarez, Daneli Ruiz Sanchez, Alejandro De Leon Cruz, Luis Angel Ceceñas Falcon, Marco Mendez Saenz, Carlos Alfredo Gomez de la Cruz, Mario Alberto Campos Coy, Juan Manuel Sánchez Castillo, Oscar Vidal Gutierrez, Joaquin Manzo Merino, Silvia Peralonso Bombin, Yuridia Evangelina Rodríguez Rosales, Gabriela Lugo Martinez, Jimena Maria Iglesias, Sebastian Medina Gonzalez, Claudia Catalina Beltran Rodriguez

**Affiliations:** 1 Oncology Service, University Hospital “Dr. Jose Eleuterio Gonzalez”, Autonomous University of Nuevo León, San Nicolás de los Garza 66455, NL, Mexico; vmoyervidesjuarez@hotmail.com (V.M.O.J.); daneli111778@hotmail.com (D.R.S.); drdeleonom@gmail.com (A.D.L.C.); manuel1572@hotmail.com (J.M.S.C.); vidal_oscar@hotmail.com (O.V.G.); 2 Pathology Department, University Hospital “Dr. Jose Eleuterio Gonzalez”, Autonomous University of Nuevo León, San Nicolás de los Garza 66455, NL, Mexico; luis.cecenas@patologiahu.com.mx; 3 Radiology and Diagnostic Imaging Department, University Hospital “Dr. Jose Eleuterio Gonzalez”, Autonomous University of Nuevo León, San Nicolás de los Garza 66455, NL, Mexico; drmarcomendez@yahoo.com (M.M.S.); calfgocru23@gmail.com (C.A.G.d.l.C.); 4 Otorhinolaryngology Service, University Hospital “Dr. Jose Eleuterio Gonzalez”, Autonomous University of Nuevo León, San Nicolás de los Garza 66455, NL, Mexico; camposcoy58@hotmail.com; 5 MSD, Ciudad de México 01090, CDMX, Mexico; silvia.peralonso1@merck.com (S.P.B.); yuridia.evangelina.rodriguez@merck.com (Y.E.R.R.); 6 Zeed, Pharmaceutical Solutions, Ciudad de México 03100, CDMX, Mexico; direccion@zeed.com.mx; 7 MSD, Bogotá 111111, Colombia; sebastian.medina.gonzalez@merck.com (S.M.G.); claudia.catalina.beltran.rodriguez@merck.com (C.C.B.R.)

**Keywords:** head and neck squamous cell carcinoma, treatment patterns, time to treatment, time to diagnosis, HPV status

## Abstract

**Objective:** In Mexico, head and neck cancers pose a significant health burden. GLOBOCAN reported approximately 3183 new cases and 1636 deaths in 2020. Despite being the sixth leading cause of cancer incidence and mortality worldwide, data on epidemiology and treatment patterns in Mexico remain limited. This study aimed to characterize the profile, clinical features, and management of patients with Stage III–IVB head and neck squamous cell carcinoma (HNSCC) in a real-world setting. **Methods:** We retrospectively analyzed a database of 187 patients with Stage III, IVA, or IVB HNSCC treated at the University Hospital Dr. José Eleuterio González. Demographics, disease characteristics, and treatment patterns were summarized as frequencies and percentages. Exploratory endpoints included clinical outcomes and recurrence types. **Results:** The cohort was 82.9% male (*n* = 155). The most frequent tumor sites were the oral cavity (36.9%) and larynx (36.9%), with 55% (*n* = 103) diagnosed at stage IVA. Of 75 cases tested for p16, 35.3% (*n* = 36) were positive. The median time from symptom onset to diagnosis was 166.5 days (95% CI: 123.4–197.8) and from diagnosis to treatment 42 days (95% CI: 31.6–50.4). Initial treatments included surgery (36.4%), chemoradiotherapy (24.6%), induction chemotherapy (19.8%), supportive care (11.2%), and radiotherapy (8%). Locoregional control was achieved in 42.8% of patients, with an overall recurrence rate of 2.8%. **Conclusions:** This study provides real-world insights into the epidemiology and management of locally advanced HNSCC in Mexico, outlining the patient journey from initial symptoms to treatment and underscoring the need for more individualized therapeutic strategies based on molecular profiling and clinical characteristics.

## 1. Introduction

Head and neck squamous cell carcinomas (HNSCCs) are a heterogeneous group of neoplasms that show diverse clinical and biological characteristics [1]. The burden of HNSCC varies across countries/regions; according to data from the Global Cancer Observatory (GLOBOCAN) 2020, head and neck cancers, including those of the lip, oral cavity, larynx, nasopharynx, oropharynx, and hypopharynx, represent nearly 880,000 new cases and 440,000 deaths worldwide per year [2]. There were approximately 3183 new cases and 1636 deaths from the disease in Mexico in 2020 []. The incidence of HNSCC continues to rise and is anticipated to increase by 30% (1.08 million new cases annually) by 2030 [3]. This disease has generally been correlated with exposure to tobacco-derived carcinogens, excessive alcohol consumption, or both [1]. However, the role of human papillomavirus (HPV), particularly HPV16, has emerged as a significant oncogenic driver, highlighting the need for biomarker-driven therapeutic strategies. [4,5].

Despite the increasing incidence of HPV-related HNSCC, data on its prevalence in Mexico remain inconsistent. A study by Carrera-Cáceres (2007) reported a prevalence of 72% in a small cohort, with 61.5% in carcinomas in the oral cavity, 74% in the larynx, and 100% in the oropharynx, and HPV-16 being the most prevalent type (47%) [6]. In contrast, Méndez-Matías (2021) identified a prevalence of 22.3% (133/368) in a Mexican population [7].

However, a 2022 study involving 414 patients with HNSCC, including cases of the oropharynx, larynx, and oral cavity, provided valuable insights into the molecular and clinical characteristics of this disease in Mexican patients. Age data were available for 365 cases, with a mean of 65.0 ± 12.8 years (range: 27–95 years); 71.3% of patients were male. Notably, 24.1% of cases were diagnosed at 55 years or younger. In terms of anatomical distribution, 19.6% of cases were located in the oropharynx, 41.2% in the oral cavity, and 39.1% in the larynx. The majority of patients (65%) were diagnosed at advanced stages (III and IV), with moderately differentiated tumors being the predominant histological grade (66.6%). Additionally, 64.4% of patients reported smoking, while 61.5% reported alcohol consumption [7].

The management of HNSCC patients with locally advanced disease usually includes a multimodal strategy with surgery, radiation therapy, and systemic therapies [8]. HNSCC of the oral cavity is generally treated with surgical resection, followed by adjuvant radiation or chemotherapy plus radiation (chemoradiation or chemoradiotherapy (CRT)), depending on the disease stage. CRT has been the primary approach to treat cancers that arise in the pharynx or larynx [1]. The epidermal growth factor receptor (EGFR; also known as HER1) monoclonal antibody cetuximab is approved by the Food and Drug Administration (FDA) as a radiation sensitizer, alone or in combination with chemotherapy [9]. Even though cisplatin is the preferred systemic agent for radio sensitization for patients with locally advanced HNSCC undergoing definitive CRT, many of these patients have relative or absolute contraindications for this treatment, including kidney dysfunction, hearing loss, neuropathy, advanced age, and performance status. For patients ineligible to receive treatment with cisplatin, alternate systemic radio-sensitizing agents include cetuximab and carboplatin-based chemotherapy. Furthermore, cetuximab and carboplatin-fluorouracil with radiotherapy (RT) have been associated with improved survival compared with RT alone in randomized clinical trials [10,11].

There are no data available about the patient journey and the clinical characteristics of HNSCC patients in Mexico, including time from the initial diagnosis to the initial treatment with curative intent, type of treatments that are received (surgery, induction, chemoradiotherapy or radiotherapy), as well as clinical outcome of these curative intention treatments. Hence, we aimed to analyze data registries from a reference institution in Northern Mexico to allow for a better understanding of the epidemiology, diagnosis, and treatment patterns of locally advanced HNSCC. Clinical outcomes, including locoregional control and recurrence, were described as exploratory objectives, without any intended statistical correlation to treatment strategies. These data will contribute to a better understanding of the opportunities for innovative therapies.

## 2. Materials and Methods

### 2.1. Study Design

This retrospective database study selected individuals with stage III-IVB HNSCC diagnosed between 1 January 2017 and 31 December 2021. The study utilized a database compiled from clinical records at the University Hospital Dr. José Eleuterio González in Monterrey, the largest cancer treatment center in Northern Mexico. The database was manually curated by the principal investigator from electronic and physical medical records of his patients, ensuring a structured dataset for analysis. Structured and unstructured data, including laboratory test results, biomarker values, and physician notes, were extracted, processed, and included in the database, which is refreshed monthly. The study aimed to evaluate patient journey metrics, including time to diagnosis (TTD) and time to treatment (TTT), as well as treatment patterns and disease progression. This study received ethical approval from the Institutional Review Board (IRB) of the University Hospital Dr. José Eleuterio González (initial approval PI22-00219, amendment EN23-00019). Given the retrospective nature of the study and the use of de-identified patient data, individual patient consent was not required, and no data were shared with third parties.

### 2.2. Study Population

Patients included in this study were diagnosed with stage III-IVB HNSCC between 1 January 2017 and 31 December 2021 with a primary tumor located in the oral cavity, oropharynx, hypopharynx, or larynx. Patients with unknown primary tumor location were included if squamous cell histology was confirmed and the clinical presentation was consistent with head and neck squamous cell carcinoma. All included patients received treatment at the University Hospital Dr. José Eleuterio González. Exclusion criteria comprised patients diagnosed with nasopharyngeal carcinoma, salivary gland carcinoma, paranasal sinus tumors, or thyroid carcinoma. Patients previously treated with anti-PD-1/PD-L1 immunotherapy were also excluded. Additionally, cases with incomplete clinical records that prevented the assessment of key study variables were not considered for analysis. The index date for each patient was defined as the date of symptom onset as recorded in the medical history. The observation period extended from the index date until the earliest occurrence of one of the following: patient death, last recorded follow-up visit, or the study cut-off date of 31 December 2022. Only patients with locally advanced HNSCC were included given the distinct treatment complexity, poorer prognosis, and higher clinical burden associated with these stages. Including earlier-stage cases would have introduced heterogeneity in treatment approaches and outcomes that fall outside the scope of this retrospective analysis.

### 2.3. Study Outcomes

Extracted variables included demographic information such as age at diagnosis, sex, smoking history, alcohol consumption history, and comorbidities. Clinical characteristics included tumor site, TNM staging at diagnosis (classified according to the 8th edition of the AJCC/UICC staging system), histopathological subtype, and HPV status when available. Treatment details were recorded, encompassing first-line therapy, including surgery, radiation therapy, and systemic therapy such as chemotherapy or targeted therapy. Disease progression was assessed based on locoregional control, recurrence, or distant metastases. Time intervals were calculated as follows: TTD was defined as the time from symptom onset to histopathological diagnosis, while TTT was measured from histopathological diagnosis to the initiation of the first treatment. In cases where treatment was initiated before histopathological confirmation, such as surgeries with subsequent biopsy confirmation, the dates of diagnosis and treatment were considered the same to ensure consistency in the dataset. In cases where the exact day of a given event was missing from records, the first day of the month was assigned to maintain temporal consistency. The follow-up period was calculated from the date of histopathological diagnosis to the date of last contact, death, or study cut-off date (31 December 2022), whichever occurred first. Median follow-up time was estimated for the overall cohort. Clinical outcomes, including disease control and recurrence, were included as exploratory objectives. No attempt was made to correlate treatment patterns with these outcomes, as the study was not designed or powered to assess treatment efficacy or long-term survival.

### 2.4. Statistical Analysis

Data were analyzed using IBM SPSS Statistics version 29.0. Descriptive statistics were used to summarize demographic and clinical characteristics. Quantitative variables were reported using means, standard deviations (SDs), medians, interquartile ranges (IQRs), and minimum/maximum values. Qualitative variables were summarized as frequencies and percentages.

## 3. Results

A total of 273 records of patients diagnosed with HNSCC between 1 January 2017 and 31 December 2021 were reviewed. After data review and validation, 187 patients (68.5%) met the study selection criteria and were included in the analysis. The average age of the cohort was 59.6 years ± 11.7 years, and 82.9% were male (*n* = 155). The mean body mass index (BMI) was 24.3 ± 5.2. Most patients reported tobacco use (61%, *n*= 114) and alcohol consumption (56.1%, *n* = 105), (Table 1).

Most of the cases were localized in the larynx (36.9%, *n* = 69) and oral cavity (36.9%, *n* = 69); and more than half of them were diagnosed in stage IVA (55.1%, *n* = 103) (Table 2). Two patients (1.1%) had tumors with unknown primary origin; these were included in the cohort based on confirmed histopathologic diagnosis of squamous cell carcinoma and treatment initiation at our center.

To assess HPV positivity, evaluation of p16 expression by immunohistochemistry was performed only when the primary tumor location was the oropharynx (including tonsils) and oral cavity (including tongue, mobile tongue, maxilla, hard palate, and upper alveolar rim). Out of the 102 cases with oral cavity and oropharynx squamous cell carcinoma, a total of 75 (73.5%) were tested for p16, of which 48% (*n* = 36) tested positive. Among the HPV-positive cases, most of them were 59 years old or younger, averaging 54 ± 10, with 58.3% (*n* = 21) localized in the oropharynx and 41.7% (*n* = 15) in the oral cavity. Regarding clinical stages at diagnosis for p16 positive cases, 41.7% (*n* = 15) were in stage IVA, followed by 30.6% (*n* = 11) in stage IVB (Table 3).

### 3.1. HNSCC Patient Journey and Treatment Flow

Regarding patient journey, the median time for a patient to receive a histopathologic diagnosis after symptoms onset was of 166.5 days (95% CI, 123.4–197.8) (≈5.5 months); and the median TTT was of 42 days (95% CI, 31.6–50.4) (≈1.4 months).

**Figure 1:**
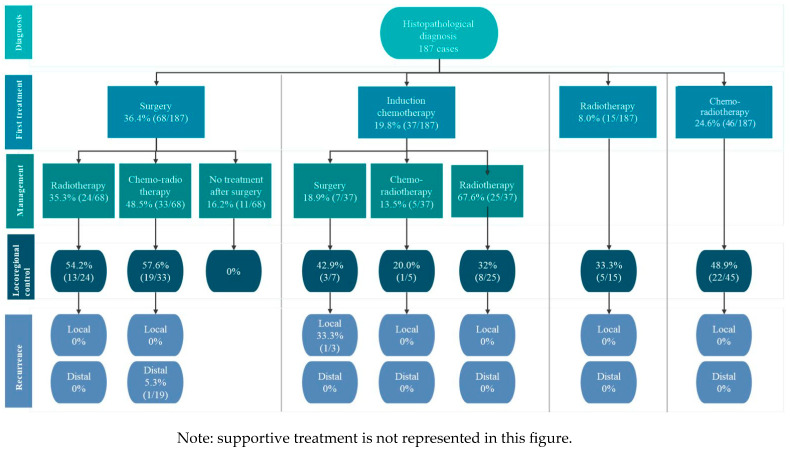
Head and neck squamous cell carcinoma patient journey.

The initial treatment that patients received after diagnosis was one of the following: surgery (*n* = 68, 36.4%), chemo-radiotherapy (*n* = 46, 24.6%), induction chemotherapy (*n* = 37, 19.8%), supportive care (*n* = 21, 11.2%), and radiotherapy (*n* = 15, 8%) (Figure 1).

The following sections describe the journey of patients after histopathologic diagnosis, according to the first treatment received.

#### 3.1.1. Flow 1: Histopathologic Diagnosis to Surgery

For the 68 patients receiving surgery as first treatment, the median time between the histopathologic diagnosis and the procedure was 21 days (95% C.I. 0–43.2) (≈0.7 months). After surgery, 24 out of 68 patients (35.3%) received radiotherapy, and more than half of them (*n* = 13/24 patients, 54.2%) reported locoregional control. None of the patients reported recurrence of the disease during the observation time. On the other hand, among the 68 patients, 33 (48.5%) underwent chemo-radiotherapy following surgery, with 57.6% (*n* = 19) achieving locoregional control and only one patient experiencing distal recurrence. This patient was managed with fluorouracil and carboplatin. The remaining patients that underwent surgery as first treatment (*n* = 11, 16.2%) did not receive any further additional treatment, and none of them reported achieving locoregional control.

#### 3.1.2. Flow 2: Histopathologic Diagnosis to Chemo-Radiotherapy

The median time between the histopathologic diagnosis to chemo-radiotherapy administration in the 46 cases with this treatment scheme was 76 days (95% I.C. 61.8–91.3) (≈2.5 months). All these patients received concurrent definitive radiotherapy. Almost half of the patients reported locoregional control (*n* = 22, 47.8%); none of the patients presented recurrence during the observation period.

#### 3.1.3. Flow 3: Histopathologic Diagnosis to Induction Chemotherapy

Of the 37 patients receiving chemotherapy induction, the median time between the histopathologic diagnosis to treatment was 44 days (95% C.I. 34–58.7) (≈1.4 months). After the induction chemotherapy, patients followed three different paths:

Chemo-radio therapy: provided to 5 patients (13.5%); one patient reported locoregional control with no disease recurrence.

Surgery: Performed in 7 patients (18.9%). Three of these patients (*n* = 3, 42.9%) reported locoregional control, and only one patient presented local recurrence that was treated with re-irradiation.

Radiotherapy: Implemented in 25 patients (67.6%). The radiotherapy received and the output was as follows: (1) 80% (*n* = 20) received concurrent definitive radiotherapy, and seven of these patients (35%) reported locoregional control; (2) 16% (*n* = 4) received adjuvant monotherapy, none of them reported locoregional control, and (3) 4% (*n* = 1) received concurrent adjuvant therapy, one reporting locoregional control. None of the patients reporting locoregional control reported recurrence of the disease.

#### 3.1.4. Flow 4: Histopathologic Diagnosis to Radiotherapy

For the 15 patients receiving radiotherapy as first treatment, the median TTT was 50 days (95% C.I. 35.9–70) (≈1.6 months). Two of these patients (13.3%) received radiotherapy as palliative treatment. Five patients (33.3%) reported locoregional control, with no recurrence reported during the observation period. Among the 166 patients who received active treatment within the cohort, 71 patients (42.8%) achieved locoregional control. During the study observation period, only 2 patients (2.8%) experienced recurrences, with one case being local and the other distal.

The median follow-up time for the overall cohort was 382 days (≈12.5 months) (95% C.I.,163–820). These outcomes reflect early post-treatment disease status and are reported as exploratory findings; the study was not designed to assess long-term survival or recurrence risk.

## 4. Discussion

This study presents an epidemiological description of Mexican patients with head and neck squamous cell carcinoma (HNSCC) in stages III, IVA, and IVB and its treatment patterns. The sociodemographic distribution in our study is similar to other studies reported in the Mexican population; nevertheless, this research adds valuable information on the patient’s journey from symptoms onset to the first treatment, and the outcome.

A large proportion of oropharyngeal and oral cavity tumors in our cohort were related to HPV infection. We observed a prevalence of p16 positivity (48%) that closely aligned with the findings in Carrera-Cáceres et al.’s study (47%) [6]. However, our results were notably higher than those reported by Mendez et al. (32%), although they included laryngeal tumors in their analysis [7]. The mean age for p16-positive patients that we found in our cohort (54 years) is similar to that reported in other studies (~53 years) [12], which is lower than that reported in the total population with HNSCC.

Although surgical resection with curative intent followed by postoperative RT has been one of the recommended treatments for most patients with locally advanced head and neck tumors, it is associated with unsatisfactory outcomes, since it is reported that patients exhibit 30% of locoregional failures, 25% of distant metastases, and 5-year survival rates of 50% [13,14]. In our cohort, nearly half of the patients (45.8%) who underwent surgery followed by radiotherapy did not achieve locoregional control. Furthermore, although the use of chemoradiotherapy (CRT) post-surgery yielded somewhat improved results (with a 42.4% failure in locoregional control), these outcomes still remain unfavorable.

These findings further underscore the urgent need for more effective treatment strategies for locally advanced HNSCC, as well as the critical importance of strengthening early detection and awareness at the primary healthcare level. Notably, our cohort exhibited a median interval of 5.5 months (166.5 days) from symptom onset to histopathologic diagnosis, highlighting a substantial delay in the diagnostic process. This diagnostic delay represents a major barrier in the management of HNSCC, as it has been associated with disease upstaging and poorer overall survival outcomes [15,16]. Such prolonged intervals may result from both patient-level factors—such as limited disease awareness, socioeconomic constraints, or fear of diagnosis—and system-level barriers including referral inefficiencies, limited availability of specialists, and delays in diagnostic imaging [17,18]. In resource-limited settings, addressing these delays is essential to improve timely diagnosis and optimize outcomes.

Furthermore, timely initiation of treatment has also been recognized as a key prognostic factor in HNSCC. A large retrospective cohort study involving 37,730 patients demonstrated that a time-to-treatment (TTT) interval exceeding 67 days after diagnosis was associated with significantly worse overall survival [19]. Similarly, other studies have identified delays beyond 60 days as independent predictors of recurrence and mortality [20]. In our study, the median TTT was 42 days, which falls below these critical thresholds. However, it is important to acknowledge that part of our study period overlapped with the COVID-19 pandemic, a factor known to contribute to healthcare delays, including cancer diagnosis and treatment initiation [21].

Previous studies have highlighted the prognostic significance of pathological subtypes and their implications for treatment selection. HPV-positive oropharyngeal squamous cell carcinomas have consistently demonstrated superior treatment responses and overall survival compared to HPV-negative tumors. A pooled analysis by O’Sullivan et al. reported that HPV-positive patients had a markedly improved prognosis, which has justified the use of de-intensified treatment regimens in this subgroup [22]. In contrast, HPV-negative tumors—particularly those arising in the oral cavity and larynx—tend to be more aggressive, less responsive to chemoradiotherapy, and are associated with significantly poorer survival [22,23]. Furthermore, histologic subtypes such as basaloid squamous cell carcinoma have been associated with a more aggressive clinical course and inferior outcomes when compared to conventional HNSCC [24]. These differences underscore the need for a pathology-driven approach to prognostication and treatment planning. There is growing interest in de-escalation strategies for HPV-positive HNSCC, motivated by its favorable prognosis; however, their efficacy remains unproven and requires validation in well-designed clinical trials. Conversely, escalation of therapy may be warranted in high-risk subgroups, such as HPV-negative or basaloid variants, which are typically associated with more aggressive disease and worse prognostic outcomes [25,26].

The study has several limitations inherently tied to its retrospective design. Being a single-center study, the sample size is limited, which impacts the generalizability of the results. Additionally, the presence of missing information and improper data registration further adds to the study’s limitations. Furthermore, clinical outcomes reported here are strictly exploratory and descriptive in nature. The study was not designed to draw causal inferences between treatments and outcomes, and the limited follow-up further restricts survival analysis. These limitations are inherent to the retrospective, real-world design and underscore the need for future prospective, multicenter studies with longer observation periods. Nonetheless, this study provides valuable insights into the epidemiology, molecular characteristics, and treatment patterns of locally advanced HNSCC in Mexico. The findings underscore the necessity for a more tailored, precision-based approach to treating these patients. Given the prevalence of HPV-positive cases, a shift toward individualized treatment regimens based on molecular markers could optimize patient outcomes.

## Figures and Tables

**Table 1:** Demographic characteristics.

	Female	Male	Overall
Patients, *n* (%)	32 (17.1)	155 (82.9)	187 (100)
Age at diagnosis, years ± SD	59.7 ± 11.9	59.6 ± 11.8	59.6 ± 11.7
BMI * at baseline, kg/m^2^ ± SD	24.3 ± 5.2	24.3 ± 5.2	24.3 ± 5.2
Tobacco use, *n* (%)	7 (21.9)	107 (69.0)	114 (61.0)
Alcohol consumption, *n* (%)	9 (28.1)	96 (62.0)	105 (56.1)
Hypertension, *n* (%)	5 (15.6)	36 (23.2)	41 (21.9)

* BMI: body mass index.

**Table 2:** Distribution of cases by site of HNSCC and clinical stage.

	Stage	III		IVA		IVB		Total	
HNSCC Site		*n*	%	*n*	%	*n*	%	*n*	%
Oropharynx		6	3.2	20	10.7	7	3.7	33	17.6
Oral cavity		13	7.0	36	19.3	20	10.7	69	36.9
Larynx		24	12.8	41	21.9	4	2.1	69	36.9
Hypopharynx		1	0.5	5	2.7	8	4.3	14	7.5
Unknown primary tumor		0	0	1	0.5	1	0.5	2	1.1
Total		44	23.5	103	55.1	40	21.4	187	100.0

**Table 3:** Distribution of cases by p16 status and clinical variables.

p-16 Test	Clinical Stage	Oropharynx		Oral Cavity		Total	
		*n*	%	*n*	%	*n*	%
Negative	III	3	37.5%	5	62.5%	8	20.5%
	IVA	6	26.1%	17	73.9%	23	59.0%
	IVB	1	12.5%	7	87.5%	8	20.5%
	**Subtotal**	**10**	**25.6%**	**29**	**74.4%**	**39**	**100.0%**
Positive	III	3	30.0%	7	70.0%	10	27.8%
	IVA	13	86.7%	2	13.3%	15	41.7%
	IVB	5	45.5%	6	54.5%	11	30.6%
	**Subtotal**	**21**	**58.3%**	**15**	**41.7%**	**36**	**100.0%**

## Data Availability

The data are owned by the principal investigator and will be shared upon request, adhering to best practices for documentation and the protection of sensitive information.

## References

[1] Johnson D.E., Burtness B., Leemans C.R., Lui V.W.Y., Bauman J.E., Grandis J.R. (2020). Head and neck squamous cell carcinoma. Nat. Rev. Dis. Primers.

[2] International Agency for Research on Cancer (2020). Global Cancer Observatory: Cancer Today.

[3] Ferlay J., Colombet M., Soerjomataram I., Mathers C., Parkin D.M., Piñeros M., Znaor A., Bray F. (2019). Estimating the global cancer incidence and mortality in 2018: GLOBOCAN sources and methods. Int. J. Cancer.

[4] Michaud D.S., Langevin S.M., Eliot M., Nelson H.H., Pawlita M., McClean M.D., Kelsey K.T. (2014). High-risk HPV types and head and neck cancer. Int. J. Cancer.

[5] Stein A.P., Saha S., Kraninger J.L., Swick A.D., Yu M., Lambert P.F., Kimple R.J. (2015). Prevalence of Human Papillomavirus in Oropharyngeal Cancer: A Systematic Review. Cancer J..

[6] Carrera-Cáceres S., Hernández-Hernández D.M., Apresa T., Gallegos-Hernández J.F., Guido-Jiménez M., García-Carrancá A. (2007). Prevalence of human Papillomavirus in head and neck cancer in Mexico: A case-control study. BMC Cancer.

[7] Méndez-Matías G., Velázquez-Velázquez C., Castro-Oropeza R., Mantilla-Morales A., Ocampo-Sandoval D., Burgos-González A., Heredia-Gutiérrez C., Alvarado-Cabrero I., Sánchez-Sandoval R., Barco-Bazán A. (2021). Prevalence of HPV in Mexican Patients with Head and Neck Squamous Carcinoma and Identification of Potential Prognostic Biomarkers. Cancers.

[8] Orlandi E., Alfieri S., Simon C., Trama A., Licitra L., Hackl M., Van Eycken E., Henau K., Dimitrova N., Sekerija M. (2019). Treatment challenges in and outside a network setting: Head and neck cancers. Eur. J. Surg. Oncol..

[9] Bonner J.A., Harari P.M., Giralt J., Azarnia N., Shin D.M., Cohen R.B., Jones C.U., Sur R., Raben D., Jassem J. (2006). Radiotherapy plus cetuximab for squamous-cell carcinoma of the head and neck. N. Engl. J. Med..

[10] Gebre-Medhin M., Brun E., Engström P., Cange H.H., Hammarstedt-Nordenvall L., Reizenstein J., Nyman J., Abel E., Friesland S., Sjödin H. (2021). ARTSCAN III: A Randomized Phase III Study Comparing Chemoradiotherapy with Cisplatin Versus Cetuximab in Patients With Locoregionally Advanced Head and Neck Squamous Cell Cancer. J. Clin. Oncol. Off. J. Am. Soc. Clin. Oncol..

[11] Gillison M.L., Trotti A.M., Harris J., Eisbruch A., Harari P.M., Adelstein D.J., Jordan R.C.K., Zhao W., Sturgis E.M., Burtness B. (2019). Radiotherapy plus cetuximab or cisplatin in human papillomavirus-positive oropharyngeal cancer (NRG Oncology RTOG 1016): A randomised, multicentre, non-inferiority trial. Lancet.

[12] Windon M.J., D’Souza G., Rettig E.M., Westra W.H., van Zante A., Wang S.J., Ryan W.R., Mydlarz W.K., Ha P.K., Miles B.A. (2018). Increasing prevalence of human papillomavirus–positive oropharyngeal cancers among older adults. Cancer.

[13] Marta G.N., Silva V., De Andrade Carvalho H., de Arruda F.F., Hanna S.A., Gadia R., da Silva J.L.F., Correa S.F.M., Vita Abreu C.E.C., Riera R. (2014). Intensity-modulated radiation therapy for head and neck cancer: Systematic review and meta-analysis. Radiother. Oncol..

[14] Marta G.N., William W.N., Feher O., Carvalho A.L., Kowalski L.P. (2015). Induction chemotherapy for oral cavity cancer patients: Current status and future perspectives. Oral Oncol..

[15] Schoonbeek R.C., de Vries J., Bras L., Sidorenkov G., Plaat B.E.C., Witjes M.J.H., van der Laan B.F.A.M., Hoek J.G.M.v.D., van Dijk B.A.C., Langendijk J.A. (2022). The effect of treatment delay on quality of life and overall survival in head and neck cancer patients. Eur. J. Cancer Care.

[16] Lauritzen B.B., Schmidt J.J., Christian G., Irene W., von Buchwald C. (2021). Impact of delay in diagnosis and treatment-initiation on disease stage and survival in oral cavity cancer: A systematic review. Acta Oncol..

[17] Chang A.K., Kruglik C.P., Valentin G.F.S., Barry M.M., Brundage W.J., Devenney B., Gagne H.M., Nelson C.J., Silverman D., Sajisevi M.B. (2024). Factors associated with radiation treatment delay in head and neck squamous cell carcinoma. J. Radiother. Pract..

[18] Lalango F., Kabagenyi F., Seguya A., Byaruhanga R., Otiti J. (2024). A descriptive study on diagnostic timelines, and factors influencing delayed diagnosis among adult head and neck cancer patients at Uganda cancer institute. World J. Surg. Oncol..

[19] Rygalski C.J., Zhao S., Eskander A., Zhan K.Y., Mroz E.A., Brock G., Silverman D.A., Blakaj D., Bonomi M.R., Carrau R.L. (2021). Time to Surgery and Survival in Head and Neck Cancer. Ann. Surg. Oncol..

[20] Liao D.Z., Schlecht N.F., Rosenblatt G., Kinkhabwala C.M., Leonard J.A., Ference R.S., Prystowsky M.B., Ow T.J., Schiff B.A., Smith R.V. (2019). Association of Delayed Time to Treatment Initiation With Overall Survival and Recurrence Among Patients with Head and Neck Squamous Cell Carcinoma in an Underserved Urban Population. JAMA Otolaryngol. Head Neck Surg..

[21] Mack D.P., Spencer H., Wang K., Lewis G.D. (2023). The Effects of the COVID-19 Pandemic on Cancer Staging in Patients Diagnosed With Head and Neck Cancer. Cureus.

[22] O’Rorke M.A., Ellison M.V., Murray L.J., Moran M., James J., Anderson L.A. (2012). Human papillomavirus related head and neck cancer survival: A systematic review and meta-analysis. Oral Oncol..

[23] Zhou C., Parsons J.L. (2020). The radiobiology of HPV-positive and HPV-negative head and neck squamous cell carcinoma. Expert Rev. Mol. Med..

[24] Linton O.R., Moore M.G., Brigance J.S., Gordon C.A., Summerlin D.-J., McDonald M.W. (2013). Prognostic significance of basaloid squamous cell carcinoma in head and neck cancer. JAMA Otolaryngol. Head Neck Surg..

[25] Cheraghlou S., Torabi S.J., Husain Z.A., Otremba M.D., Osborn H.A., Mehra S., Yarbrough W.G., Burtness B.A., Judson B.L. (2019). HPV status in unknown primary head and neck cancer: Prognosis and treatment outcomes. Laryngoscope.

[26] Rosenberg A.J., Vokes E.E. (2021). Optimizing Treatment De-Escalation in Head and Neck Cancer: Current and Future Perspectives. Oncologist.

